# Biofilms and device-associated infections.

**DOI:** 10.3201/eid0702.010226

**Published:** 2001

**Authors:** R M Donlan

**Affiliations:** Centers for Disease Control and Prevention Atlanta, Georgia 30333, USA. rld8@cdc.gov

## Abstract

Microorganisms commonly attach to living and nonliving surfaces, including those of indwelling medical devices, and form biofilms made up of extracellular polymers. In this state, microorganisms are highly resistant to antimicrobial treatment and are tenaciously bound to the surface. To better understand and control biofilms on indwelling medical devices, researchers should develop reliable sampling and measurement techniques, investigate the role of biofilms in antimicrobial drug resistance, and establish the link between biofilm contamination and patient infection.


### Biofilms and Device-Associated Infections

**Rodney M. Donlan**
Centers for Disease Control and Prevention Atlanta, Georgia, USA

Microorganisms commonly attach to living and nonliving surfaces, including those of indwelling medical devices, and form biofilms made up of extracellular polymers. In this state, microorganisms are highly resistant to antimicrobial treatment and are tenaciously bound to the surface. To better understand and control biofilms on indwelling medical devices, researchers should develop reliable sampling and measurement techniques, investigate the role of biofilms in antimicrobial drug resistance, and establish the link between biofilm contamination and patient infection.

Microbial biofilms develop when microorganisms irreversibly adhere to a submerged surface and produce extracellular polymers that facilitate adhesion and provide a structural matrix. This surface may be inert, nonliving material or living tissue. Biofilm-associated microorganisms behave differently from planktonic (freely suspended) organisms with respect to growth rates and ability to resist antimicrobial treatments and therefore pose a public health problem. This article describes the microbial biofilms that develop on or within indwelling medical devices (e.g., contact lenses, central venous catheters and needleless connectors, endotracheal tubes, intrauterine devices, mechanical heart valves, pacemakers, peritoneal dialysis catheters, prosthetic joints, tympanostomy tubes, urinary catheters, and voice prostheses).

### Characteristics of Biofilms on Indwelling Medical Devices

Biofilms on indwelling medical devices may be composed of gram-positive or gram-negative bacteria or yeasts. Bacteria commonly isolated from these devices include the gram-positive *Enterococcus faecalis, Staphylococcus aureus, Staphylococcus epidermidis*, and *Streptococcus* viridans; and the gram-negative *Escherichia coli, Klebsiella pneumoniae, Proteus mirabilis,* and *Pseudomonas aeruginosa*. These organisms may originate from the skin of patients or health-care workers, tap water to which entry ports are exposed, or other sources in the environment. Biofilms may be composed of a single species or multiple species, depending on the device and its duration of use in the patient. Urinary catheter biofilms may initially be composed of single species, but longer exposures inevitably lead to multispecies biofilms (1). A distinguishing characteristic of biofilms is the presence of extracellular polymeric substances, primarily polysaccharides, surrounding and encasing the cells. These polysaccharides, which have been visualized by scanning electron microscopy (Figure 1), appear either as thin strands connecting the cells to the surface and one another or as sheets of amorphous material on a surface. Most biofilm volume is actually composed of this extracellular polymeric substance rather than cells, a fact that has been confirmed by ruthenium red staining and transmission electron microscopy (2). This biofilm matrix may act as a filter, entrapping minerals (1) or host-produced serum components (3). Biofilms are both tenacious and highly resistant to antimicrobial treatment; Anwar et al. (4) showed that treatment with levels of tobramycin far in excess of the MIC reduced biofilm cell counts for *P. aeruginosa* by approximately 2 logs, while the same dosage provided a >8-log decrease in planktonic cells of this organism.

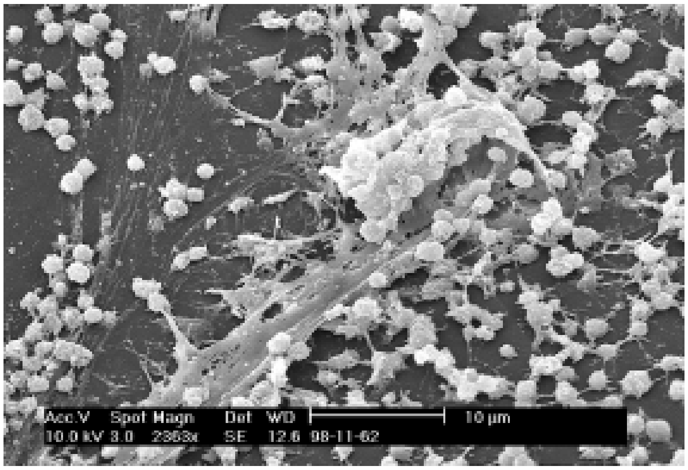

Figure 1. Scanning electron micrograph of a *Staphylococcus* biofilm on the inner surface of a needleless connector.
Photograph by Janice Carr, Centers for Disease Control and Prevention, Atlanta, GA USA.

### Factors Influencing Rate and Extent of Biofilm Formation

When an indwelling medical device is contaminated with microorganisms, several variables determine whether a biofilm develops. First the microorganisms must adhere to the exposed surfaces of the device long enough to become irreversibly attached. The rate of cell attachment depends on the number and types of cells in the liquid to which the device is exposed, the flow rate of liquid through the device, and the physicochemical characteristics of the surface. Components in the liquid may alter the surface properties and also affect rate of attachment. Once these cells irreversibly attach and

Address for correspondence: Rodney M. Donlan, National Center for Infectious Diseases, Hospital Infections Program, Centers for Disease Control and Prevention, 1600 Clifton Road, Mailstop C16, Atlanta, GA 30333, USA; fax: 404-639-2322; e-mail: rld8@cdc.gov produce extracellular polysaccharides to develop a biofilm, rate of growth is influenced by flow rate, nutrient composition of the medium, antimicrobial-drug concentration, and ambient temperature. These factors can be illustrated by examining what is known about biofilms on three types of indwelling medical devices: central venous catheters, mechanical heart valves, and urinary (Foley) catheters.

### Central Venous Catheter Biofilms

Scanning and transmission electron microscopy has shown that virtually all indwelling central venous catheters are colonized by microorganisms embedded in a biofilm matrix (5). The organisms most commonly isolated from catheter biofilms are *Staphylococcus epidermidis, S. aureus, Candida albicans, P. aeruginosa, K. pneumoniae,* and *Enterococcus faecalis* (6,7).

These organisms originate from patient's skin microflora, exogenous microflora from health-care personnel, or contaminated infusates. They gain access to the catheter by migration externally from the skin along the exterior catheter surface or internally from the catheter hub or port (8). Colonization of these devices can occur rapidly (within 24 hours) and may be a function of host-produced conditioning films (platelets, plasma, and tissue proteins) (8). Raad et al. (9) found that biofilm formation on central venous catheters was universal, but the extent and location of biofilm formation depended on the duration of catheterization: short-term (<10 days) catheters had greater biofilm formation on the external surface; long-term catheters ( 30 days) had more biofilm formation on the catheter inner lumen. The nature of the fluid administered through central venous catheters may affect microbial growth: gram-positive organisms (*S. epidermidis, S. aureus)* did not grow well in intravenous fluids, whereas the gram-negative aquatic organisms (e.g., *P. aeruginosa, Klebsiella* spp*., Enterobacter* spp*., Serratia* spp*.,* and *Pantoea* sp*.)* sustained growth (10-14). Because many of these solutions have limited nutrients, bacterial growth rarely produces turbidity, meaning that numbers are $<10^7$ organisms per milliliter. The number of organisms on the catheter tip is related to occurrence of bloodstream infection in the patient (7,15-17), supporting the concept of a critical level of biofilm development above which substantial cell detachment and embolism occur.

Several studies have examined the effect of various types of antimicrobial treatment in controlling biofilm formation on these devices. Freeman and Gould (18) found that addition of sodium metabisulfite to the dextrose-heparin flush of the left atrial catheter eliminated microbial colonization of these catheters. Darouiche et al. (19) found that catheters impregnated with minocycline and rifampin were less likely to be colonized than those impregnated with chlorhexidine and silver sulfadiazine. In a study by Kamal et al. (20), catheters coated with a cationic surfactant (tridodecylmethylammonium chloride), which was in turn used to bond cephalosporin to the surface, were less likely to become contaminated and develop biofilms than were untreated catheters. Flowers et al. (21) found that an attachable subcutaneous cuff containing silver ions inserted after local application of polyantibiotic ointment conferred a protective effect on catheters, resulting in lower rates of contamination. Maki (8) suggested several ways to control biofilms on central venous catheters, including using aseptic technique during implantation, using topical antibiotics, minimizing the duration of catheterization, using an in-line filter for intravenous fluids, creating a mechanical barrier to prevent influx of organisms by attaching the catheter to a surgically implanted cuff, coating the inner lumen of the catheter with an antimicrobial agent, and removing the contaminated device.

### Mechanical Heart Valve Biofilms

Microorganisms may attach and develop biofilms on components of mechanical heart valves and surrounding tissues of the heart, leading to a condition known as prosthetic valve endocarditis. The primary organisms responsible for this condition are *S. epidermidis, S. aureus, Streptococcus* spp*.,* gram-negative bacilli, diphtheroids, enterococci, and *Candida* spp. These organisms may originate from the skin, other indwelling devices such as central venous catheters, or dental work (3). The identity of the causative microorganism is related to its source: whether the contaminating organism originated at the time of surgery (early endocarditis, usually caused by *S. epidermidis*), from an invasive procedure such as dental work (*Streptococcus spp.),* or from an indwelling device (a variety of organisms). Implantation of the mechanical heart valve causes tissue damage, and circulating platelets and fibrin tend to accumulate where the valve has been attached. Microorganisms also have a greater tendency to colonize these locations (3). The resulting biofilms more commonly develop on the tissue surrounding the prosthesis or the sewing cuff fabric used to attach the device to the tissue (22,23) than on the valve itself (24). Antimicrobial agents are usually administered during valve replacement and whenever the patient has dental work to prevent initial attachment by killing all microorganisms introduced into the bloodstream. As with biofilms on other indwelling devices, relatively few patients can be cured of a biofilm infection by antibiotic therapy alone (25). Illingworth et al. (22) found that a silver-coated sewing cuff on a St. Jude mechanical heart valve (St. Jude Medical Inc., St. Paul, MN) implanted into a guinea pig artificially infected with *S. epidermidis* produced less inflammation than did uncoated fabric. Although the number of attached organisms was not determined, the authors concluded that the degree of inflammation was proportional to the number of viable organisms. Carrel et al. (23) also found this approach was effective in in vitro studies with different organisms.

### Urinary Catheter Biofilms

Urinary catheters are tubular latex or silicone devices, which when inserted may readily acquire biofilms on the inner or outer surfaces. The organisms commonly contaminating these devices and developing biofilms are *S. epidermidis, Enterococcus faecalis, E. coli, Proteus mirabilis, P. aeruginosa, K. pneumoniae,* and other gram-negative organisms (1). The longer the urinary catheter remains in place, the greater the tendency of these organisms to develop biofilms and result in urinary tract infections. For example, 10% to 50% of patients undergoing short-term urinary catheterization ( 7 days) but virtually all patients undergoing long-term catheterization (>28 days) become infected (1). Brisset et al. (26) found that adhesion to catheter materials was dependent on the hydrophobicity of both the organisms and the surfaces; catheters displaying both hydrophobic and hydrophilic regions allowed colonization of the widest variety of organisms. Divalent cations (calcium and magnesium) and increase in urinary pH and ionic strength all resulted in an increase in bacterial attachment. Tunney et al. (27) stated that no single material is more effective in preventing colonization, including silicone, polyurethane, composite biomaterials, or hydrogel-coated materials. Certain component organisms of these biofilms produce urease, which hydrolyzes the urea in the patient's urine to ammonium hydroxide. The elevated pH that results at the biofilm-urine interface results in precipitation of minerals such as struvite and hydroxyapatite. These mineral-containing biofilms form encrustations that may completely block the inner lumen of the catheter (27). Bacteria may ascend the inner lumen into the patient's bladder in 1 to 3 days (28); this rate may be influenced by the presence of swarming organisms such as *Proteus* spp. (D. Stickler, pers. comm.). Several strategies have been attempted to control urinary catheter biofilms: antimicrobial ointments and lubricants, bladder instillation or irrigation, antimicrobial agents in collection bags, impregnation of the catheter with antimicrobial agents such as silver oxide, or use of systemic antibiotics (29). Most such strategies have been ineffective, although silver-impregnated catheters delayed onset of bacteriuria for up to 4 days. In a rabbit model, biofilms on Foley catheter surfaces were highly resistant to high levels of amdinocillin, a beta-lactam antibiotic (30). However, Stickler et al. (31) found that treatment of a patient with a polymicrobial biofilm-infected catheter with ciprofloxacin allowed the catheter to clear and provide uninterrupted drainage for 10 weeks. Morris et al. (32) found that time to blockage of catheters in a laboratory model system was shortest for hydrogel- or silver-coated latex catheters and longest for an Eschmann Folatex S All Silicone catheter (Portex Ltd., Hythe, Kent, England). Biofilms of several gram-negative organisms were reduced by exposure to mandelic acid plus lactic acid (33). In a study in which ciprofloxacin-containing liposomes were coated onto a hydrogel-containing Foley catheter and exposed in a rabbit model, the time to development of bacteriuria was double that with untreated catheters, although infection ultimately occurred in the rabbits with treated catheters (34).

### Directions for Future Research

To better understand and control biofilms on indwelling medical devices, research must progress in several key areas. More reliable techniques for collecting and measuring biofilms should be developed. For central venous catheters, the reference method for quantification of biofilms on catheter tips is the roll-plate technique, in which the tip of the catheter is removed and rolled over the surface of a nonselective medium. Quantification of the biofilm depends on the number of organisms recovered by contact with the agar surface. Biofilm-associated cells on the inner lumen of the device are not detected with this method, which has low diagnostic sensitivity and low predictive value for catheter-related bacteremia (7). In addition, this method cannot detect more than 1,000 colony-forming units (CFU) per tip. A method that used sonication plus vortexing as a means of quantifying biofilms on catheter tips showed that a level of $10^4$ CFU per tip is predictive of catheter-related septicemia. Although this method is an improvement over the semi-quantitative roll-plate technique, the recovery efficiency of the method needs to be determined (i.e., the percentage of cells that are not recovered and quantified). Zufferey et al. (35) described a method for rapidly detecting biofilm cells on catheters by direct staining of the catheter with acridine orange. Although they found good agreement with culture techniques and noted that this technique provided more rapid results, they did not quantify cells; instead, they recorded a simple positive or negative result. Techniques that allow counting of biofilm cells directly on the catheter surface would be an improvement over established methods.

Model systems should be developed and used to study biofilm processes on various indwelling medical devices. These systems should closely simulate the in vivo or in situ conditions for each device, while at the same time providing reproducible, accurate results. To investigate biofilm formation on needleless connectors, Donlan et al. (14) used a biofilm disk reactor system (Figure 2) that incorporated a medium (intravenous fluid), a material (teflon coupons or needleless connectors), an organism (*Enterobacter cloacae*), and a flow rate (1 mL/min) that closely simulated conditions of use for these devices. Results were both reproducible and precise, and the system was capable of developing a steady state biofilm (Figure 3). This system design could be used to investigate and compare various biofilm control treatments, device design modifications, or different media formulations. By performing a similar experiment in an animal model system, biofilm processes in vivo could be predicted.

Another area of great importance from a public health perspective is the role of biofilms in antimicrobial-drug resistance. Bacteria within biofilms are intrinsically more

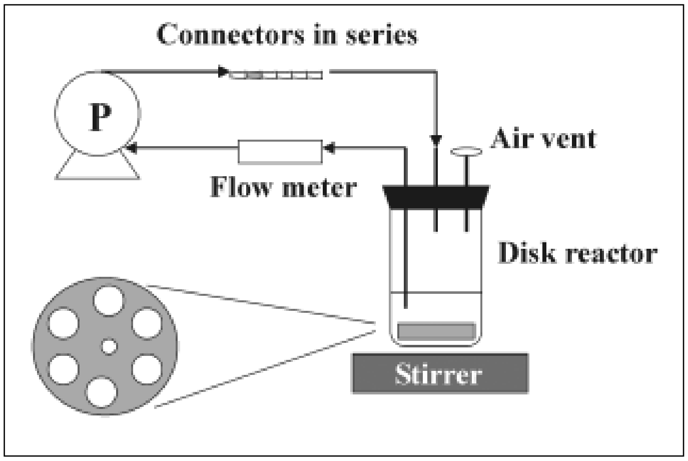

Figure 2. Biofilm disk reactor system.

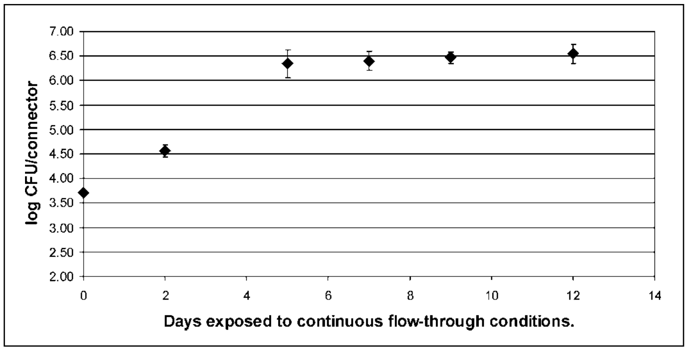

Figure 3. *Enterobacter cloacae* biofilm formation on needleless connectors.

resistant to antimicrobial agents than planktonic cells because of the diminished rates of mass transport of antimicrobial molecules to the biofilm associated cells (36) or because biofilm cells differ physiologically from planktonic cells (37). Antimicrobial concentrations sufficient to inactivate planktonic organisms are generally inadequate to inactivate biofilm organisms, especially those deep within the biofilm, potentially selecting for resistant subpopulations. This selection may have implications for treatments that use controlled release of antimicrobial agents to prevent biofilm growth on indwelling devices. Bacteria can transfer extachromosomal genetic elements within biofilms; Roberts et al. (38) demonstrated transfer of a conjugative transposon in a model oral biofilm. Hausner and Wuertz (39) demonstrated conjugation in a lab-grown biofilm with rates one to three orders of magnitude higher than those obtained by classic plating techniques. Resistance-plasmids could also be transferred within biofilms on indwelling medical devices.

The link between biofilm contamination of an indwelling device and patient infection is often unclear. Raad et al. (9) noted that biofilm formation was universal on vascular catheters collected from patients, yet observed that this universal colonization rarely resulted in bloodstream infection. A better understanding of the factors that control cell detachment may help answer the questions: Is there a critical biofilm density threshold above which detachment occurs? What is the role of the exopolymers in this process? Davies et al. (40) demonstrated the role of acyl homoserine lactones (HSL) in biofilms of *P. aeruginosa* and showed that HSL-knockouts were deficient in biofilm architecture and much more readily detached than wild-type organisms. Stickler et al. (41) detected these quorum-sensing molecules in biofilms on urethral catheters. A greater understanding of cell-to-cell communication within biofilms may lead to better predictability of biofilm processes such as detachment, as well as more effective control strategies.

### Conclusions

Microbial biofilms may pose a public health problem for persons requiring indwelling medical devices. The microorganisms in biofilms are difficult or impossible to treat with antimicrobial agents; detachment from the device may result in infection. Although medical devices may differ widely in design and use characteristics, specific factors determine susceptibility of a device to microbial contamination and biofilm formation. For example, duration of use, number and type of organisms to which the device is exposed, flow rate and composition of the medium in or on the device, device material construction, and conditioning films on the device all may influence biofilm formation. More effective biofilm control strategies should result as researchers develop more reliable techniques for measuring biofilms and better model systems for evaluating control strategies. A clearer picture of the importance of biofilms in public health should also result as the role of biofilms in antimicrobial-drug resistance is investigated and the link is established between biofilm contamination and patient infection.

Dr. Donlan is team leader for the Division of Healthcare Quality Promotion Biofilm Laboratory, National Center for Infectious Diseases, CDC. His research interests focus on biofilms on indwelling medical devices, the role of biofilms in antimicrobial-drug resistance, and survival and treatment of pathogenic organisms in potable water system biofilms.

### References

1. Stickler DJ. Bacterial biofilms and the encrustation of urethral catheters. Biofouling 1996;94:293-305.
2. Jones HC, Roth IL, Saunders WM III. Electron microscopic study of a slime layer. J Bacteriol 1969;99:316-25.
3. Braunwald E. Valvular heart disease. In: Braunwald E, editor. Heart disease. 5th ed. Vol. 2. Philadelphia: W.B. Saunders Co.; 1997. p. 1007-66.
4. Anwar H, Strap JL, Chen K, Costerton JW. Dynamic interactions of biofilms of mucoid *Pseudomonas aeruginosa* with tobramycin and piperacillin. Antimicrob Agents Chemother 1992;36:1208-14.
5. Raad I. Intravascular-catheter-related infections. Lancet 1998;351:893-8.
6. Elliott TSJ, Moss HA, Tebbs SE, Wilson IC, Bonser RS, Graham TR, et al. Novel approach to investigate a source of microbial contamination of central venous catheters. Eur J Clin Microbiol Infect Dis 1997;16:210-3.
7. Raad II, Sabbagh MF, Rand KH, Sherertz RJ. Quantitative tip culture methods and the diagnosis of central venous catheter-related infections. Diagn Microbiol Infect Dis 1992;15:13-20.
8. Maki DG. Infections caused by intravascular devices used for infusion therapy: pathogenesis, prevention, and management. In: Bisno AL, Waldovogel FA, editors. Infections associated with indwelling medical devices. 2nd ed. Washington: American Society for Microbiology; 1994. p. 155-212.
9. Raad I, Costerton W, Sabharwal U, Sacilowski M, Anaissie W, Bodey G. Ultrastructural analysis of indwelling vascular catheters: a quantitative relationship between luminal colonization and duration of placement. J Infect Dis 1993;168:400-7.
10. Maki DG, Mermel LA. Infections due to infusion therapy. In: Bennett JV, Brachman PS, editors. Hospital infections. 4th ed. Philadelphia: Lippincott-Raven; 1998. p. 689-724.
11. Maki DG, Martin WT. Nationwide epidemic of septicemia caused by contaminated infusion products. IV. Growth of microbial pathogens in fluids for intravenous infusion. J Infect Dis 1975;131:267-72.
12. Anderson RL, Highsmith AK, Holland BW. Comparison of the standard pour plate procedure and the ATP and Limulus Amoebocyte Lysate procedures for the detection of microbial contamination in intravenous fluids. J Clin Microbiol 1986;23:465-8.
13. Failla ML, Benedict CD, Weinberg ED. Bacterial and fungal growth in total parenteral nutrition solutions. Antonie Van Leeuwenhoek 1975;41:319-28.
14. Donlan R, Murga R, Carson L. Growing biofilms in intravenous fluids. In: Wimpenny J, Gilbert P, Walker J, Brading M, Bayston R, editors. Biofilms, the good, the bad, and the ugly. Presented at the fourth meeting of the Biofilm Club; 1999; Powys, UK. p. 23-9.
15. Aufwerber E, Ringertz S, Ransjo U. Routine semiquantitative cultures and central venous catheter-related bacteremia. APMIS 1991;99:627-30.
16. Corona ML, Peters SG, Narr BJ, Thompson RL. Subspecialty clinics: critical care medicine. Infections related to central venous catheters. Mayo Clin Proc 1990;65:979-86.
17. Anaissie E, Samonis G, Kontoyiannis D, Costerton J, Sabharwal U, Bodey G, et al. Role of catheter colonization and infrequent hematogenous seeding in catheter-related infections. Eur J Clin Microbiol Infect Dis 1995;14:135-7.
18. Freeman R, Gould FK. Infection and intravascular catheters [letter]. J Antimicrob Chemother 1985;15:258.
19. Darouiche RO, Raad II, Heard SO, Thornby JI, Wenker OC, Gabrielli A, et al. A comparison of two antimicrobial-impregnated central venous catheters. N Engl J Med 1999;340:1-8.
20. Kamal GD, Pfaller MA, Rempe LE, Jebson PJR. Reduced intravascular catheter infection by antibiotic bonding. A prospective, randomized, controlled trial. JAMA 1991;265:2364-8.

21. Flowers RH, Schwenzer KJ, Kopel RF, Fisch MJ, Tucker SI, Farr BM. Efficacy of an attachable subcutaneous cuff for the prevention of intravascular catheter-related infection. JAMA 1989;261:878-83.
22. Illingworth BL, Tweden K, Schroeder RF, Cameron JD. In vivo efficacy of silver-coated (Silzone) infection-resistant polyester fabric against a biofilm-producing bacteria, *Staphylococcus epidermidis*. J Heart Valve Dis 1998;7:524-30.
23. Carrel T, Nguyen T, Kipfer B, Althaus U. Definitive cure of recurrent prosthetic endocarditis using silver-coated St. Jude medical heart valves: a preliminary case report. J Heart Valve Dis 1998;7:531-3.
24. Karchmer AW, Gibbons GW. Infections of prosthetic heart valves and vascular grafts. In: Bisno AL, Waldovogel FA, editors. Infections associated with indwelling medical devices. 2nd ed. Washington: American Society for Microbiology; 1994. p. 213-49.
25. Hancock EW. Artificial valve disease. In: Schlant RC, Alexander RW, editors. The heart arteries and veins. New York: McGraw-Hill, Inc.; 1994. p. 1539-45.
26. Brisset L, Vernet-Garnier V, Carquin J, Burde A, Flament JB, Choisy C. In vivo and in vitro analysis of the ability of urinary catheters to microbial colonization. Pathol Biol 1996;44:397-404.
27. Tunney MM, Jones DS, Gorman SP. Biofilm and biofilm-related encrustation of urinary tract devices. In: Doyle RJ, editor. Methods in enzymology. San Diego: Academic Press; 1999. p. 558-66.
28. McLean RJC, Nickel JC, Olson ME. Biofilm associated urinary tract infections. In: Lappin-Scott HM, Costerton JW, editors. Microbial biofilms. Cambridge: Cambridge University Press; 1995. p. 261-73.
29. Kaye D, Hessen MT. Infections associated with foreign bodies in the urinary tract. In: Bisno AL, Waldovogel FA, editors. Infections associated with indwelling medical devices. 2nd ed. Washington: American Society for Microbiology; 1994. p. 291-307.
30. Olson ME, Nickel JC, Khoury AE, Morck DW, Cleeland R, Costerton JW. Amdinocillin treatment of catheter-associated bacteriuria in rabbits. J Infect Dis 1989;159:1065-72.
31. Stickler DJ, King J, Nettleton J, Winters C. The structure of urinary catheter encrusting bacterial biofilms. Cells and Materials 1993;3:315-9.
32. Morris NS, Stickler DJ, Winters C. Which indwelling urethral catheters resist encrustation by *Proteus mirabilis* biofilms. Br J Urol 1997;80:58-63.
33. Stickler D, Hewett P. Activity of antiseptics against biofilms of mixed bacterial species growing on silicone surfaces. Eur J Clin Microbiol Infect Dis 1991;10:416-21.
34. Pugach JL, Ditizio V, Mittelman MW, Bruce AW, Dicosmo F, Khoury AE. Antibiotic hydrogel coated Foley catheters for prevention of urinary tract infection in a rabbit model. J Urol 1999;162:883-7.
35. Zufferey J, Rime B, Francioli P, Bille J. Simple method for rapid diagnosis of catheter-associated infection by direct acridine orange staining of catheter tips. J Clin Microbiol 1988;26:175-7.
36. Suci PA, Mittelman MW, Yu FP, Geesey GG. Investigation of ciprofloxacin penetration into *Pseudomonas aeruginosa* biofilms. Antimicrob Agents Chemother 1994;38:2125-33.
37. Evans DJ, Allison DG, Brown MRW, Gilbert P. Susceptibility of *Pseudomonas aeruginosa* and *Escherichia coli* biofilms towards ciprofloxacin: effect of specific growth rate. J Antimicrob Chemother 1991;27:177-84.
38. Roberts AP, Pratten J, Wilson M, Mullany P. Transfer of a conjugative transposon, Tn5397, in a model oral biofilm. FEMS Microbiol Lett 1999;177:63-6.
39. Hausner M, Wuertz S. High rates of conjugation in bacterial biofilms as determined by quantitative in situ analysis. Appl Environ Microbiol 1999;65:3710-3.
40. Davies DG, Parsek MR, Pearson JP, Iglewski BH, Costerton JW, Greenberg EP. The involvement of cell-to-cell signals in the development of a bacterial biofilm. Science 1998;280:295-8.
41. Stickler DJ, Morris NS, McLean RJC, Fuqua C. Biofilms on indwelling urethral catheters produce quorum-sensing signal molecules in situ and in vitro. Appl Environ Microbiol 1998;64:3486-90.
